# A comparative study of religious beliefs, spiritual intelligence and spiritual well-being in two therapies based on education (anonymous drug user) and methadone in drug user in Iran

**DOI:** 10.1186/s13104-023-06377-0

**Published:** 2023-06-12

**Authors:** Raheleh Rajabi, Hassan Eslami Aliabadi, Mohammad Javad Mahdizadeh, Mansooreh Azzizadeh Forouzi

**Affiliations:** 1 Department of Nursing, Zarand School of Nursing, Kerman University of Medical Sciences, Kerman, Iran; 2 Nursing midwifery school, Assistant Professor of Nursing, Department of Nursing, Ferdows School of Health and Allied Medical Sciences, Birjand university of Medical Sciences, Birjand, Iran; 3 Instructor of Nursing, Nursing Department, Zarand School of Nursing, Kerman University of Medical Sciences, Kerman, Iran; 4 Medical surgical nursing, Neuroscience Research Center, Institute of Neuropharmacology, Kerman University of Medical Sciences, Kerman, Iran

**Keywords:** Religious beliefs, Spiritual intelligence, Spiritual well-being, Anonymous, Drug user, Methadone

## Abstract

**Objective:**

Prevention of high-risk behaviors has always been considered; According to the researches, a person’s religious attitudes and beliefs and intelligence quotient are associated with the prevention of high-risk behaviors such as drug addiction, and religiosity and spirituality also reduce drug addiction; Therefore, this study was conducted to compare religious beliefs, intelligence and spiritual health in two methods of education-based treatment and methadone in people with addiction.

**Results:**

A comparative study was performed among 184 people on all drug users admitted to these wards that treated with methadone and participants of meetings of anonymous drug users. Four questionnaires were used to collect information. Mean, and standard deviation were used to describe the demographic characteristics of participants. Chi-square and fisher tests were used to compare demographic information in the two groups. The present study was performed following acquisition of the code of ethics (IR.BUMS.REC.1395.156) from Research Ethics Committee of Birjand University of Medical Sciences.

The results showed that the frequency distribution of gender, job status and education status in the two groups were significantly different from each other (p < 0.05). The results showed that the mean score of spiritual intelligence in the group of anonymous drug user is significantly higher than the methadone group (p = 0.001). The results showed that the mean of spiritual well-being and religious belief in the group of anonymous drug user was significantly higher than the methadone group (p < 0.05); Therefore, health care providers should try to strengthen this area of people’s lives in families and society through educational programs.

## Introduction

Substance abuse is the fourth crisis after the nuclear crisis, the increase in population and environmental pollution [1]. Addiction is the most challenging issue among governments and an economic, social, health and security issue among countries [2]. The World Health Organization (WHO) annual report indicates about 200 million drug users in the world; 8.2% of the Iranian people suffer from drug use disorders, while it is 2% in Kazakhstan and 2.2% in Russia [3, 4]. In Iran, the growth rate of drug abuse has been more than three times the population growth rate over the past 20 years [5]. Recurrence of addiction occur in almost 80% of people who withdraw from addiction in the first 6 months. Thus, addiction treatment programs should not only target the reduction and cessation of drug use, but should also consider important psychological variables associated with the onset and continuation of drug use [6].

Drug abuse is the beginning of other forms of crime, including theft, trafficking, immoral offenses and is a social crisis for all societies, so addiction prevention policy is mandatory [7]. Iran-Afghanistan border and drug transit from Iran have predisposed to drug trafficking and the domestic consumer market has grown rapidly due to its easy accessibility [8]. Complementary interventions such as psychotherapy, group therapy, occupational therapy, exercise therapy, faith therapy, family therapy, and medication reduce the recurrence from 25 to 2% [9].

Many drug users are unable to abstain permanently despite various treatments. Methadone maintenance therapy prevents drug relapse and improves the patient’s mental and physical quality. Methadone can control the physical and psychological conditions of drug users until they are able to successfully detoxify [10–12]. The Association of Narcotics Anonymous has considered community-based treatment method and tries to change people’s life styles through the support of peers and helps them to communicate with others and the world consciously and effectively [13–15].

The Association of Narcotics Anonymous uses twelve steps in three dimensions: self-help programs, social support, and spiritual strengthening [16]. Studies have shown that religion has a positive effect on the healing process [17]. Martyr Motahhari believed that religion was one of the main pillars of culture of any nation and an important indicator of mental health [18]. Loyal believers feel less abandoned and alone, and avoid using drugs. Parents are the role models of family members, so they should prioritize life skills training from the smallest to the most complex ones [19].

Biological, psychological, social and spiritual needs of human beings should be met [20]. Spirituality is one of the effective factors in preventing drug abuse [21] and it covers all areas of health at all ages [22]. According to Elkins, genuine spirituality is love and affection for all beings [23]. There is a positive relationship between physical health, spiritual health, meaning of life, and pray [24, 25].

A series of studies believed that IQ and EQ were not responsible for everything and were not really effective, and introduced a third intelligence called spiritual quotient (SQ) [26].

Spiritual intelligence is awareness of the world and the situation of human in the world and appears when we live with complete spirituality [27]. Drigas defines spiritual intelligence as one’s ability to behave intellectually and compassionately, while maintaining inner and outer peace regardless of circumstances [28]. Saleh Moghadam et al. evaluated quality of life in three groups of narcotics anonymous, community-based and methadone maintenance therapies, and showed that narcotics anonymous received higher scores in all aspects of quality of life than the other two groups [29].

Active participation of drug users in the Association of Narcotics Anonymous and adherence to the 12 principles improved their social and religious activities, as well as their quality of life [30, 31].

We can find a correlation between one’s religious attitude and beliefs, IQ, and prevention of high-risk behaviors such as drug abuse. As there was lack of information about the difference in the religious and spiritual characteristics between the two groups and the cultural and religious differences in the Iranian society, we decided to compare religious beliefs, intelligence and spiritual health in two treatment methods.

## Methods

### Study design

A comparative study was performed on all drug users undergoing methadone treatment and narcotics anonymous.

### Participants

#### Eligibility criteria for participants

The research settings were Imam Reza and Shahid Chamran hospitals and the association of narcotics anonymous in Birjand and Ferdows cities in southeastern Iran. Inclusion criteria included educated patients diagnosed with substance dependence, who were willing to participate in the study, had no physical or mental illness, did not take medicine or receive medical interventions for any reason, while exclusion criteria included reluctance to continue the study and incomplete questionnaires.

### Data collection tools

Demographic information form and three questionnaires were used to collect information. Demographic and background information included age, sex, marital status, employment, level of education, family history, duration of substance use, economic level, and the cause of drug use.

Spiritual intelligence scale.

This questionnaire designed and validated by Ali Badi et al. in Iran [32] consisted of 42 items with 4 subscales: general thoughts and belief (12 items), ability to confront and interact with problems (15 items), attend to ethical issues (8 items), self – consciousness, love and interest (7 items). The items were rated on a five-point Likert scale from strongly agree to strongly disagree. The score of second scale was scored inversed. Cronbach’s alpha was used to determine the reliability of the spiritual intelligence scale (85%). The validity of the questionnaire, its score correlates with the score criterion question and indicate that there is a positive relationship between them.( r = 55%, p = 0.0001) which shows that spiritual intelligence questionnaire is valid [33]. In the present study, ten experts confirmed the validity of this questionnaire as well as its reliability using Cronbach’s alpha (0.87).

### Spiritual well-being scale

The spiritual well-being scale is a 20-item self-report instrument with two subscales. The religious well-being subscale (10 items) assesses the vertical dimension of spirituality, while the existential well-being subscale (10 items) measures the horizontal dimension of well-being in relation to the world around us, including a sense of life purpose and life satisfaction. The scale is published in Paloutzian and Ellison [34] and Ellison [35]. Each item is rated on a six-point Likert scale from strongly agree to strongly disagree, with no mid-point. About half of the items are reverse worded to minimize the role of response sets. The scores for the Existential Well-being (EWB) and Religious Well-being (RWB) subscales range between 10 and 60. Therefore, the total score of the SWBS is from 20 to 120. We categorized the scores of the Spiritual Well-being Scale (SWBS) as low [20–40], moderate (41–99), and high (100–120). For the RWB subscale, a score of 10–20 reflects unsatisfactory relationship with God, while scores 21–49 and 50–60 reflect moderate and positive relationship with God, respectively. For the EWB subscale, “low satisfaction with life”, “relative lack of clarity about purpose in life”, and “moderate and high level of satisfaction and purpose in life” were considered, respectively [36].

Allah Bakhshian et al. translated this scale into Persian and determined the validity of this questionnaire by content validity and its reliability by Cronbach’s alpha coefficient of 0.82 [37]. In the present study, ten experts confirmed the validity of this questionnaire as well as its total reliability coefficient of 0.83 through Cronbach’s alpha.

### The duke university religion index (DUREL)

The five-item DUREL has three dimensions: organizational religiosity (one item), non-organizational religiosity (one item), and intrinsic religiosity (three items). Organizational religiosity and non-organizational religiosity are scored on a six-point Likert-type scale, while intrinsic religiosity is scored one a five-point Likert-type scale. The total score is calculated by summing the sores of all items ranging from 5 to 27, but the authors do not recommend summing all three subscales because combining all three subscales could result in subscale scores canceling out the effects of each other [38]. Safari et al. in Iran confirmed its validity using convergent validity and its reliability using test-retest and Cronbach’s alpha coefficient of 0.86–0.92 [39]. In the present study, ten experts confirmed the validity of this questionnaire and its total reliability coefficient of 0.89 using Cronbach’s alpha.

### Statistical **methods**

Descriptive statistics (frequency, percentage, mean, and standard deviation) were used to describe the demographic characteristics of participants. Chi-square and fisher’s exact tests were used to compare demographic information in the two groups. According to the normality, independent t-test was used to compare the mean studied variables in the two groups, while Pearson correlation coefficient test was used to examine the relationship between variables. A significance level of 0.05 was considered. SPSS 19 was used for data analysis.

### Sample size

The pilot study was used to estimate the sample size in the present study, so 20 participants were selected. Then, based on the results of the pilot study, the type I error of 5%, power of 80% and the correlation coefficient of 0.29, 184 people were included in two groups randomly. The 95% confidence coefficient was calculated, so the confidence interval was 1.96.

## Results

This study was performed on those undergoing methadone treatment vs. narcotics anonymous. The results also showed a significant difference in the frequency distribution of gender, job and education level between the two groups (p < 0.05) (Table 1).

**Table 1:** Comparison of the frequency distribution of demographic information in the two groups of methadone and narcotics anonymous

Variable	Methadone group Frequency (percentage)	Narcotics Anonymous group Frequency(percentage)	P- Value
Sex			
Male	89(100)	76(80)	P < 0.001
Female	0(0)	19(20)	
Marital status			
Married	62(69.7)	62(65.3)	0.4* P = 0.52
Single	27(30.3)	33(34.7)	
Economic situation			
Weak	23(25.8)	23(24.2)	6.45* P = 0.09
Medium	36(40.4)	54(56.8)	
Higher than average	16(18)	9(9.5)	
Good	14(15.7)	9(9.5)	
Family history			
Yes	45(50.6)	49(51.6)	0.02* P = 0.89
No	44(49.4)	46(48.4)	
Employment			
Unemployed	21(23.6)	22(23.2)	11.21* P = 0.01
Employed	13(14.6)	6(6.3)	
Self-Employed	49(55.1)	67(70.5)	
Retired	6(6.7)	0(0)	
Education level			
Elementary	16(18)	20(21.1)	33.12* P < 0.001
Lower secondary	17(19.1)	49(51.6)	
Diploma	37(41.6)	24(25.3)	
Bachelor’s and higher	19(21.3)	2(2.2)	

=* χ2

The mean scores of spiritual intelligences in the groups of methadone and narcotics anonymous were 96.44 ± 15.40 and 104.38 ± 13.90, respectively. The results of independent t-test showed that the mean score of spiritual intelligence in the group of narcotics anonymous was significantly higher than that in the methadone group (p = 0.001). The mean scores of spiritual well-being and religious belief in the methadone group were 76.87 ± 7.29 and 17.04 ± 5.57, respectively.

The results of independent t-test showed that the mean spiritual well-being and religious belief in the group of narcotics anonymous was significantly higher than that in the methadone group (p < 0.05) (Table 2).

**Table 2:** Comparison of mean spiritual intelligence, spiritual well-being and religious belief in two groups of methadone and narcotics anonymous

Variable	Methadone group Mean ± Standard deviation	Narcotics Anonymous group Mean ± Standard deviation	P-Value, t-test result
Spiritual intelligence scale	96.44 ± 15.40	104.38 ± 13.90	p = 0.001, t = 3.65
Spiritual well-being scale	76.87 ± 7.29	85.44 ± 8.68	p = 0.001, t = 6.84
religious beliefs	17.04 ± 5.57	18.97 ± 4.36	p = 0.009, t = 2.66

## Discussion

The present study showed that lower number of women referred to addiction treatment centers than men. Due to the social and cultural restrictions for Iranian women, so they visit addiction treatment centers less [40]. The high preponderance of addiction cases in those with high school, or lower education, is similar to other studies)[41].

Most of the participants had self-employed jobs. Karari et al. showed that self-employed low-income individuals were the greatest drug users (43.4%), followed by unemployed individuals (21.9%). The high prevalence of addiction in the low-income population and unemployed people in our study may be due to minimally available entertainment, poor culture, lack of hope and economical poverty, and the use of opiates as pain-killers [42].

However, the level of spiritual intelligence, spiritual well-being scale and religious beliefs in the narcotics anonymous group was higher than that of methadone users. Spirituality can increase individuals’ resistance against problems; Shalchi and Sohrabi indicated that spiritual intelligence had a positive relationship with increased resilience of drug users [43]. Smith also believed that spiritual intelligence played a key role in preventing substance abuse disorders because both intelligence and spirituality allowed individuals to change and perceive the situation as much as possible [44].

The studies showed that the recovery process of clients participating in the Association of Narcotics Anonymous (NA) was according to 12 steps and three dimensions (self-help programs, social support, and spiritual empowerment).

Galanter et al. reported that adaptation in the method of narcotics anonymous was very similar to the nature of Islam and Iranian culture of families (mutual assistance, hospitality and service) [45] because social support and spiritual strengthening were the main dimensions of this treatment. Therefore, there is a strong correlation between clients’ social activities (talking with each other and gaining experience from others) and their spiritual intelligence, spiritual health and religious beliefs, so participation in such social activities strengthens spirituality and quality of life. Given that clients undergoing methadone treatment have no social interaction, their spiritual intelligence, spiritual health and religious beliefs are weaker than those in the Association of Narcotics Anonymous [46]. In general, communication and interaction with other people is effective in maintaining and promoting spiritual health[47].

We found that spiritual intelligence, spiritual health and religious beliefs were moderate in drug users undergoing methadone treatment and narcotics anonymous, which is consistent with other studies [48, 49]. Iran is a religious society so that people from childhood learn religious teachings in their families and schools and become committed to it. Addiction increases a person’s need and dependence on a substance and also affects the power of human comprehension and may weaken these religious beliefs, but it cannot completely remove them from the essence of a religious person. As the clients of the present study were quitting addiction, the interventions made during the withdrawal period could also increase their ability and motivation to quit. Therefore, moderate levels of spiritual health, spiritual intelligence and spiritual beliefs are justifiable; perhaps this level of spirituality encouraged them to quit addiction in order to save themselves and their families from this devastating calamity.

Noormohammadi et al. indicated a strong association between the return of drug users and a decrease in spiritual well-being; spiritually weak people had a greater tendency to re-experience drugs. Adherence to religion and spirituality creates responsibility and commitment in individuals, and prevents them to return to drug use again [50]. In general, production a social wealth within a group is kind of return or reconstruction of lost wealth of individuals in society. This wealth has been created by members of anonymous drug users groups, which leads to quitting and continuing to quit addiction[46].

## Conclusion

One of the three main dimensions of the 12-step process of the Narcotics Anonymous (NA) is the spiritual dimension, so we found that participants in the Association of Narcotics Anonymous had higher spiritual intelligence, spiritual health, and religious beliefs than those undergoing the methadone treatment. Further studies are necessary to examine whether the level of spirituality of the people in the present study is influenced by their religious cultural context or their participation in meetings. It is necessary to strengthen this approach in the activities of non-governmental organizations.

### Limitations

Several limitations of our study are as follows: Some drug users refused to fill in the questionnaires, which caused a prolonged data collection. Conduction of a study on a population in a specific region can reduce its generalizability, so it is recommended to conduct more studies in other Iranian regions in the future.

## Data Availability

Not applicable.
